# Lipid Abnormalities and Cardiometabolic Risk in Patients with Overt and Subclinical Thyroid Disease

**DOI:** 10.1155/2011/575840

**Published:** 2011-07-18

**Authors:** Melpomeni Peppa, Grigoria Betsi, George Dimitriadis

**Affiliations:** ^1^Endocrine Unit, Second Department of Internal Medicine-Propaedeutic, Research Institute and Diabetes Center, Athens University Medical School, Attikon University Hospital, Athens, Greece; ^2^Second Department of Internal Medicine-Propaedeutic, Research Institute and Diabetes Center, Athens University Medical School, Attikon University Hospital, Athens, Greece

## Abstract

Dyslipidemia is a common finding in patients with thyroid disease, explained by the adverse effects of thyroid hormones in almost all steps of lipid metabolism. Not only overt but also subclinical hypo- and hyperthyroidism, through different mechanisms, are associated with lipid alterations, mainly concerning total and LDL cholesterol and less often HDL cholesterol, triglycerides, lipoprotein (a), apolipoprotein A1, and apolipoprotein B. In addition to quantitative, qualitative alterations of lipids have been also reported, including atherogenic and oxidized LDL and HDL particles. In thyroid disease, dyslipidemia coexists with various metabolic abnormalities and induce insulin resistance and oxidative stress via a vice-vicious cycle. The above associations in combination with the thyroid hormone induced hemodynamic alterations, might explain the increased risk of coronary artery disease, cerebral ischemia risk, and angina pectoris in older, and possibly ischemic stroke in younger patients with overt or subclinical hyperthyroidism.

## 1. Introduction

Thyroid disease, namely hypothyroidism and hyperthyroidism, constitutes the most common endocrine abnormality in recent years, diagnosed either in subclinical or clinical form. According to the 6-year duration NHANES III Study, the prevalence of hypothyroidism was 4.6% (0.3% clinical and 4.3% subclinical) and of hyperthyroidism 1.3% (0.5% clinical and 0.7% subclinical), in population aged at least 12 years, showing an age and sex dependence [1].

Thyroid disease is associated with various metabolic abnormalities, due to the effects of thyroid hormones on nearly all major metabolic pathways. Thyroid hormones regulate the basal energy expenditure through their effect on protein, carbohydrate, and lipid metabolism. This might be a direct effect or an indirect effect by modification of other regulatory hormones such as insulin or catecholamines [2]. Dyslipidemia is a common metabolic abnormality in patients with thyroid disease, either in the overt or subclinical forms of the disease, and constitutes the end result of the effect of thyroid hormones in all aspects of lipid metabolism leading to various quantitative and/or qualitative changes of triglycerides, phospholipids, cholesterol, and other lipoproteins [3].

In thyroid disease, dyslipidemia and the coexisting metabolic abnormalities, in combination with the thyroid hormone-induced hemodynamic alterations, explain the high risk for cardiovascular disease [4–7].

## 2. Effects of Thyroid Hormones on Lipid Metabolism

Thyroid hormones influence all aspects of lipid metabolism including synthesis, mobilization, and degradation [3]. Thyroid hormones stimulate cholesterol synthesis by inducing 3-hydroxy-3-methyl-glutaryl coenzyme A reductase in the liver [8]. Thyroid hormones affect lipoprotein lipase activity and thus, the hydrolysis of triglycerides into very-low, density lipoprotein (VLDL) and chylomicrons into fatty acids and glycerol [3]. In hypothyroidism, lipoprotein lipase activity in the adipose tissue has been found normal or decreased, in addition to decreased hepatic lipase activity resulting in normal or high levels of triglycerides [9–11]. In hyperthyroidism, although lipoprotein lipase activity is usually normal [10, 12], an increased liver fatty acid synthesis and oxidation is observed due to enhanced acetyl-CoA carboxylase 1 and carnitine palmitoyltransferase Ia expression leading to increased VLDL biosynthesis [13, 14]. Thyroid hormones affect cholesteryl ester transfer protein and hepatic lipase activity, which are increased in hyperthyroidism and decreased in hypothyroidism, with consequent changes not only in total high-density lipoprotein (HDL) but also in HDL subfraction levels [12, 15]. Furthermore, thyroid hormones, by binding to the thyroid hormone receptor, inhibit through a competitive action the liver X-receptor-mediated ATP-binding cassette transporter A1 gene expression, resulting in decreased HDL levels in patients with hyperthyroidism and increased in hypothyroidism [14, 16]. Experimental evidence suggests that thyroid hormones might also affect cholesterol-7alpha-hydroxylase in liver [17, 18]. Thyroid hormones, especially triiodothyronine (T3), induce low-density lipoprotein (LDL) receptor gene expression in the liver, enhancing LDL clearance and explaining the decreased or increased LDL levels observed in hyperthyroidism and hypothyroidism, respectively [3]. Thyroid receptors seem to mediate the effects of thyroid hormones on lipid metabolism, and more specifically alpha 1 receptors control the lipogenesis in white adipose tissue, and *β* receptors regulate the activity of lipogenic and lipolytic enzymes in the liver [3, 14]. The changes induced by thyroid hormones in enzyme activities, transfer proteins, and liver receptors involved in lipid metabolism are summarized in Table 1.

Dyslipidemia is also due to the coexisting metabolic abnormalities in thyroid disease including oxidative stress and insulin resistance, which induce further or aggravate the existed dyslipidemia, via a vice-vicious cycle [19–28].

The lipid abnormalities observed in thyroid disorders are presented in Table 2.

## 3. Lipid Abnormalities in Hypothyroidism

Dyslipidemia is a common finding in patients with clinical hypothyroidism, consisting of high levels of total and LDL cholesterol [3, 28–30]. Data regarding triglycerides, lipoprotein (a) (Lp(a)), HDL, apolipoprotein B (apoB), and apolipoprotein A1 (apoA1) components are scarce, reporting either higher or similar to euthyroid subjects levels [3, 22, 28–34] (Table 2). Qualitative changes of various lipid components have also been reported in clinical hypothyroidism, such as the enhanced LDL oxidation, reflected on the increased levels of markers of lipid peroxidation, such as MDA and thiobarbituric acid-reactive substances [19–24, 35].

The observed abnormalities in total and LDL cholesterol are associated with the changes in the thyroid hormone levels in hypothyroidism, as they are significantly improved after thyroxine replacement treatment [29, 30, 36–40]. However, triglycerides, apoB, apoA1, Lp(a) levels, and qualitative abnormalities might be normalized or remained unchanged after treatment, suggesting a more complex cause of dyslipidemia in hypothyroidism [29, 31, 36–43].

Subclinical hypothyroidism is also associated with lipid abnormalities, including mainly increased total and LDL cholesterol in most [3, 28–30, 44–53], but not all [54–57], studies. In contrast, HDL, triglycerides, Lp(a), apoB, and apoA1 levels did not exhibit any difference between patients with subclinical hypothyroidism and controls in the majority [29, 44–57], but not all [33, 44–48, 50, 55, 58–61], studies. Rondeau et al. found that TSH was negatively correlated with HDL-C in euthyroid overweight or obese postmenopausal women [62]. A quite recent study showed that transfer of triglycerides to HDL and phospholipids was lower in patients with subclinical hypothyroidism than that in controls while transfer of free and esterified cholesterol to HDL, HDL particle size, and paraoxonase 1 activity did not exhibit any difference [63] (Table 2).

Regarding the effects of treatment of subclinical hypothyroidism, the majority of studies suggest a normalization of total and LDL cholesterol levels after thyroxine substitution therapy [28, 30, 40–43, 48, 49, 52, 53, 64–67]. However, there are few trials where LDL did not significantly fall after treatment [29, 46, 51], especially if the pretreatment TSH levels were less than 10 mIU/L [49, 68]. Triglycerides, HDL, apoA1, apoB, and Lp(a) levels are less influenced by thyroxine treatment in the majority of studies in subclinical hypothyroidism [28–30, 38, 43–49, 52, 53, 58, 65, 66]. However, few studies exist that showed improved HDL, apoA1, apoB, and/or Lp(a) levels after treatment of subclinical hypothyroidism [46–48, 51, 52, 58, 61, 67]. A quite recent study showed that the reduced transfer of triglycerides to HDL and phospholipids in subclinical hypothyroidism was fully reversed by achievement of euthyroidism [69].

## 4. Lipid Abnormalities in Hyperthyroidism

Most of the existing studies support lower total and LDL cholesterol levels in patients with hyperthyroidism [3, 32, 39, 68–73], while only a few data support no change [21]. Lower triglycerides, HDL, apoA1, apoB, and Lp(a) levels have been found in patients with hyperthyroidism compared with euthyroid controls, which is questionable by other reports [21–23, 32, 68–72] (Table 2).

In hyperthyroidism, qualitative lipid changes, including increased levels of oxidized LDL, higher contents of thiobarbituric acid-reactive substances and dienes in LDL, low paraoxonase activity in HDL particles, and lower LDL content in antioxidant vitamin E and *β*-carotene have been found [21, 23, 24, 35]. 

The impact of treatment of hyperthyroidism on the lipid levels is not clear. Treatment with antithyroid drugs has been associated with elevated total and LDL cholesterol levels in some but not all studies [12, 21, 22, 68, 74–76]. Triglycerides are not affected by antithyroid treatment [21, 42, 70, 74, 76]. HDL, apoB, apoA1, Lp(a) levels have been found increased or unchanged after treatment [21, 22, 31, 42, 70–76].

The issue of lipid abnormalities in patients with subclinical hyperthyroidism has not been fully addressed. The existing data support normal levels of total LDL and HDL cholesterol, triglycerides, Lp(a), apoA1 and apoB while lower total and LDL cholesterol hav also e been reported [77, 78].

## 5. Thyroid Disease and Cardiovascular Risk

Most of the existing data supporting that thyroid disease is associated with increased cardiovascular risk which is mainly attributed to hemodynamic alterations as well as to a high risk of atherosclerosis [4–7].

### 5.1. Thyroid Disorders and Hemodynamic Changes

In hypothyroidism, the main functional cardiovascular disturbances involve decreased heart rate, elevated peripheral vascular resistance, increased diastolic blood pressure and cardiac afterload, reduced blood volume and cardiac preload, and diminished cardiac output. Impaired left ventricular systolic contractility at least during exercise and delayed left ventricular diastolic relaxation at rest and during exercise are common in both overt and subclinical hypothyroidism. Hypothyroidism is also associated with diastolic heart failure in the elderly [4, 7].

In hyperthyroidism, hemodynamic changes result mainly from increased *β*1-adrenergic activity. Increased triiodothyronine levels exert positive inotropic and chronotropic effects, leading to enhanced heart rate and systolic contractility and, consequently, increased cardiac output. Increased triiodothyronine stimulates sarcoplasmic reticulum Ca-ATPase, leading to systolic and diastolic dysfunction. Moreover, triiodothyronine reduces peripheral vascular resistance, causing a decrease in diastolic blood pressure and cardiac afterload, which further raises cardiac output. Decreased vascular resistance accounts for activation of renin-angiotensin-aldosterone system, which increases blood volume and cardiac preload, augmenting cardiac output even more [5, 7]. Biondi et al. found that even patients with subclinical hyperthyroidism had significantly higher average heart rate, enhanced systolic function, impaired diastolic function with prolonged isovolumic relaxation time, and increased left ventricular mass compared with euthyroid subjects [79].

### 5.2. Thyroid Disease and Atherosclerosis

As mentioned above, thyroid disease is related to the development of dyslipidemia which is a well-known atherogenic factor. Dyslipidemia induces insulin resistance oxidative stress, via a vice-vicious cycle [19–24, 35]. Insulin resistance, hypertension, inflammation, oxidative stress, and coagulation deficits are also promoted by thyroid disease, independently of dyslipidemia [4–7]. The above associations support a multifactorial origin of atherosclerosis in thyroid disease, with dyslipidemia playing an important role [4–7]. 

Overt hypothyroidism has been associated with diastolic hypertension [4, 6, 7, 80, 81] and hyperhomocysteinemia [82–85]. Increased levels of high-sensitivity C-reactive protein and coagulation deficits have been reported in patients with hypothyroidism [83, 86–88]. Higher levels of homeostasis model assessment and lower levels of Matsuda indexes, suggesting insulin resistance, have been found in patients with overt hypothyroidism compared with euthyroid subjects in some [25, 26, 89, 90] but not all [91, 92] studies. Impaired intracellular glucose catabolism and GLUT4 translocation, decreased glycogen synthesis and glucose oxidation, and altered blood flow, have been proposed as underlying mechanisms [25, 26, 90, 93, 94]. Increased intima-media thickness of the common carotid artery has been reported in some studies in patients with overt hypothyroidism [53, 95, 96]. A higher frequency and/or severity of coronary heart disease [4, 97, 98] and an increased ischemic stroke risk [99] have been reported in patients with overt hypothyroidism.

Subclinical hypothyroidism has been also associated with diastolic hypertension in most but not all studies [48, 60, 89, 100–103]. A few but not all studies have reported hyperhomocysteinemia [48, 83, 104–108], higher but also normal levels of high-sensitivity C-reactive protein [56, 57, 83, 104, 107, 109–111], and possible coagulation deficits [86, 87] in patients with subclinical hypothyroidism. Higher levels of homeostasis model assessment and lower levels of Matsuda indexes, suggesting insulin resistance, have been found in patients with subclinical hypothyroidism in some but not all studies [25, 26, 89, 104, 111]. Increased intima-media thickness of the common carotid artery has been found in some studies in subclinical hypothyroidism [45, 53]. In the Whickham Survey, an association was found between incident coronary heart disease and related mortality in patients with subclinical hypothyroidism over the 20 yrs of followup, which was attenuated after levothyroxine treatment [112]. In support of this, 3 meta-analyses suggested that subclinical hypothyroidism is associated with a significant risk of coronary heart disease and cardiovascular mortality [113–115]. Another meta-analysis by Razvi et al. showed that the incidence and prevalence of coronary heart disease and the risk of cardiovascular mortality were higher in subclinical hypothyroidism, in patients younger than 65 years old and more prevalent in women [116]. Subclinical hypothyroidism has been associated with cerebral ischemia [117]. Although Jeong et al. in a study of 382 patients with ischemic stroke found no difference in the prevalence of subclinical hypothyroidism (4,5%) compared to the general population, low-normal free T4 levels in euthyroid patients were independently associated with a higher percentage of ischemic stroke [118]. In addition, it has been demonstrated that patients with subclinical hypothyroidism (especially those with TSH ≥ 10 *μ*U/mL) and acute ischemic stroke exhibited a better level of consciousness, a milder neurological deficit at presentation, and more favorable outcomes on the 30th and 90th day compared with euthyroid patients [119, 120]. However, Rodondi et al. found no association between subclinical hypothyroidism and risk for stroke [121]. 

On the other hand, clinical hyperthyroidism has been associated with systolic hypertension, increased pulse pressure, and possibly hyperhomocysteinemia [6, 7, 122, 123] Additionally, patients with overt hyperthyroidism have a hypercoagulable state and an increased risk of thrombosis [86]. Higher levels of homeostasis model assessment and lower levels of Matsuda indexes have been reported, suggesting insulin resistance. [25, 27, 71, 91–93] Decreased fractional postprandial glucose uptake in adipose tissue, increased fasting lipolysis, increased interleukin 6, and tumour necrosis factor alpha may be associated to its development [25, 93, 124, 125]. Angina pectoris is a frequent disorder, especially in older patients with hyperthyroidism and underlying cardiac disease, and is due to increased heart rate and contractility and high myocardial oxygen demand [5]. Some cases of patients with hyperthyroidism due to Graves' disease presenting with coronary artery spasm have been reported [126–128]. Hyperthyroidism has been associated with a higher risk for ischemic stroke among young adults during a 5-year followup which was probably associated with atrial fibrillation (AF), hypercoagulability and rarely antiphospholipid antibody syndrome [129–132]. 

Subclinical hyperthyroidism has been also associated with hypertension in some but not all studies [102, 103, 133]. Higher HOMA, lower Matsuda indexes [27], and increased carotid intima thickness [134] have been found in patients with subclinical hyperthyroidism. However, the association of subclinical hyperthyroidism with coronary heart disease risk and cardiovascular mortality is still unclear. Ochs et al. found a possible association, while the meta-analysis by Singh et al. found no significant association [113, 115]. Jeong et al. in a study of 382 patients with ischemic stroke found no difference in the prevalence of subclinical hyperthyroidism (1,6%) compared to the general population [118].

## 6. Conclusion

Thyroid hormones regulate the expression of enzymes involved in all steps of lipid metabolism leading to the development of qualitative and quantitative changes of lipids, in thyroid disease. Dyslipidemia coexists with other metabolic abnormalities, including, hypertension, insulin resistance, and oxidative stress, all of them being risk factors for cardiovascular disease. In addition, dyslipidemia induces insulin resistance and oxidative stress, via a vice-vicious cycle. The existing data support that there is an increased cardiovascular morbidity in patients with thyroid disease and possibly mortality which is in part mediated by the dyslipidemia or the dyslipidemia-induced metabolic abnormalities. However, more studies need to be done, especially prospective, to elucidate the real significance of dyslipidemia or other metabolic changes to CVD morbidity and mortality in clinical and, even more, in subclinical thyroid disease.

## Figures and Tables

**Table 1:** Changes in enzyme activities, transfer proteins, and receptors involved in lipid metabolism induced by thyroid hormones.

Enzymes, transfer proteins, and liver receptors	Thyroid hormone effect
3-hydroxy-3-methyl-glutaryl coenzyme A reductase	↑
Adipose lipoprotein lipase	Usually normal (may be ↓ in hypothyroidism)
Hepatic lipase	↑
Cholesteryl ester transfer protein	↑
ATP-binding cassette transporter A1	↓
Acetyl-CoA carboxylase 1	↑
Carnitine palmitoyltransferase Ia	↑
7alpha-hydroxylase	↓
LDL receptor	↑

**Table 2:** Lipid abnormalities in clinical and subclinical thyroid disorders.

	Clinical hypothyroidism	Subclinical hypothyroidism	Clinical hyperthyroidism	Subclinical hyperthyroidism
Total cholesterol	Increased	Increased or unaltered	Decreased (rarely unaltered)	Decreased or unaltered
LDL	Increased	Increased (rarely unaltered)	Decreased (rarely unaltered)	Decreased or unaltered
HDL	Unaltered or increased	Unaltered (rarely decreased)	Unaltered or decreased	Unaltered? (a few data exist)
Lipoprotein (a)	Unaltered or increased	Unaltered (rarely increased)	Decreased? (a few data exist)	Unaltered? (a few data exist)
Triglycerides	Unaltered or increased	Unaltered (rarely increased)	Unaltered (rarely decreased)	Unaltered? (a few data exist)
Apolipoprotein B	Unaltered or increased	Unaltered or increased	Decreased? (a few data exist)	Unaltered? (a few data exist)
Apolipoprotein A1	Unaltered or increased	Unaltered (usually)	Unaltered or decreased	Unaltered? (a few data exist)

## References

[1] Hollowell JG, Staehling NW, Flanders WD (2002). Serum TSH, T4, and thyroid antibodies in the United States population (1988 to 1994): National Health and Nutrition Examination Survey (NHANES III). *Journal of Clinical Endocrinology and Metabolism*.

[2] Kim B (2008). Thyroid hormone as a determinant of energy expenditure and the basal metabolic rate. *Thyroid*.

[3] Zhu X, Cheng SY (2010). New insights into regulation of lipid metabolism by thyroid hormone. *Current Opinion in Endocrinology, Diabetes and Obesity*.

[4] Biondi B, Klein I (2004). Hypothyroidism as a risk factor for cardiovascular disease. *Endocrine*.

[5] Biondi B, Kahaly GJ (2010). Cardiovascular involvement in patients with different causes of hyperthyroidism. *Nature Reviews Endocrinology*.

[6] Klein I, Ojamaa K (2001). Thyroid hormone and the cardiovascular system. *The New England Journal of Medicine*.

[7] Fazio S, Palmieri EA, Lombardi G, Biondi B (2004). Effects of thyroid hormone on the cardiovascular system. *Recent Progress in Hormone Research*.

[8] Cachefo ANA, Boucher P, Vidon C, Dusserre E, Diraison F, Beylot M (2001). Hepatic lipogenesis and cholesterol synthesis in hyperthyroid patients. *Journal of Clinical Endocrinology and Metabolism*.

[9] Abrams JJ, Grundy SM, Ginsberg H (1981). Metabolism of plasma triglycerides in hypothyroidism and hyperthyroidism in man. *Journal of Lipid Research*.

[10] Lam KSL, Chan MK, Yeung RTT (1986). High-density lipoprotein cholesterol, hepatic lipase and lipoprotein lipase activities in thyroid dysfunction—effects of treatment. *Quarterly Journal of Medicine*.

[11] Krauss RM, Levy RI, Fredrickson DS (1974). Selective measurement of two lipase activities in postheparin plasma from normal subjects and patients with hyperlipoproteinemia. *Journal of Clinical Investigation*.

[12] Tan KCB, Shiu SWM, Kung AWC (1998). Effect of thyroid dysfunction on high-density lipoprotein subfraction metabolism: roles of hepatic lipase and cholesteryl ester transfer protein. *Journal of Clinical Endocrinology and Metabolism*.

[13] Heimberg M, Olubadewo JO, Wilcox HG (1985). Plasma lipoproteins and regulation of hepatic metabolism of fatty acids in altered thyroid states. *Endocrine Reviews*.

[14] Liu YY, Brent GA (2010). Thyroid hormone crosstalk with nuclear receptor signaling in metabolic regulation. *Trends in Endocrinology and Metabolism*.

[15] Berti JA, Amaral MEC, Boschero AC (2001). Thyroid hormone increases plasma cholesteryl ester transfer protein activity and plasma high-density lipoprotein removal rate in transgenic mice. *Metabolism*.

[16] Tancevski I, Wehinger A, Demetz E (2008). Reduced plasma high-density lipoprotein cholesterol in hyperthyroid mice coincides with decreased hepatic adenosine 5′-triphosphate-binding cassette transporter 1 expression. *Endocrinology*.

[17] Sauter G, Weiss M, Hoermann R (1997). Cholesterol 7*α*-hydroxylase activity in hypothyroidism and hyperthyroidism in humans. *Hormone and Metabolic Research*.

[18] Drover VAB, Agellon LB (2004). Regulation of the human cholesterol 7*α*-hydroxylase gene (CYP7A1) by thyroid hormone in transgenic mice. *Endocrinology*.

[19] Santi A, Duarte MMMF, Moresco RN (2010). Association between thyroid hormones, lipids and oxidative stress biomarkers in overt hypothyroidism. *Clinical Chemistry and Laboratory Medicine*.

[20] Nanda N, Bobby Z, Hamide A (2008). Association of thyroid stimulating hormone and coronary lipid risk factors with lipid peroxidation in hypothyroidism. *Clinical Chemistry and Laboratory Medicine*.

[21] Yavuz DG, Yüksel M, Deyneli O, Ozen Y, Aydin H, Akalin S (2004). Association of serum paraoxonase activity with insulin sensitivity and oxidative stress in hyperthyroid and TSH-suppressed nodular goitre patients. *Clinical Endocrinology*.

[22] Sundaram V, Hanna AN, Koneru L, Newman HAI, Falko JM (1997). Both hypothyroidism and hyperthyroidism enhance low density lipoprotein oxidation. *Journal of Clinical Endocrinology and Metabolism*.

[23] Costantini F, Pierdomenico SD, De Cesare D (1998). Effect of thyroid function on LDL oxidation. *Arteriosclerosis, Thrombosis, and Vascular Biology*.

[24] Diekman T, Demacker PNM, Kastelein JJP, Stalenhoef AFH, Wiersinga WM (1998). Increased oxidizability of low-density lipoproteins in hypothyroidism. *Journal of Clinical Endocrinology and Metabolism*.

[25] Peppa M, Koliaki C, Nikolopoulos P, Raptis SA (2010). Skeletal muscle insulin resistance in endocrine disease. *Journal of Biomedicine and Biotechnology*.

[26] Maratou E, Hadjidakis DJ, Kollias A (2009). Studies of insulin resistance in patients with clinical and subclinical hypothyroidism. *European Journal of Endocrinology*.

[27] Maratou E, Hadjidakis DJ, Peppa M (2010). Studies of insulin resistance in patients with clinical and subclinical hyperthyroidism. *European Journal of Endocrinology*.

[28] Tagami T, Tamanaha T, Shimazu S (2010). Lipid profiles in the untreated patients with Hashimoto thyroiditis and the effects of thyroxine treatment on subclinical hypothyroidism with Hashimoto thyroiditis. *Endocrine Journal*.

[29] Tzotzas T, Krassas GE, Konstantinidis T, Bougoulia M (2000). Changes in lipoprotein(a) levels in overt and subclinical hypothyroidism before and during treatment. *Thyroid*.

[30] Teixeira PDFDS, Reuters VS, Ferreira MM (2008). Lipid profile in different degrees of hypothyroidism and effects of levothyroxine replacement in mild thyroid failure. *Translational Research*.

[31] de Bruin TWA, Van Barlingen H, Van Linde-Sibenius Trip M, Van Vuurst de Vries ARR, Akveld MJ, Erkelens DW (1993). Lipoprotein(a) and apolipoprotein B plasma concentrations in hypothyroid, euthyroid, and hyperthyroid subjects. *Journal of Clinical Endocrinology and Metabolism*.

[32] Raziel A, Rosenzweig B, Botvinic V (1982). The influence of thyroid function on serum lipid profile. *Atherosclerosis*.

[33] Staub JJ, Althaus BU, Engler H (1992). Spectrum of subclinical and overt hypothyroidism: effect on thyrotropin, prolactin, and thyroid reserve, and metabolic impact on peripheral target tissues. *American Journal of Medicine*.

[34] Dullaart RPF, van Doormaal JJ, Hoogenberg K, Sluiter WJ (1995). Triiodothyronine rapidly lowers plasma lipoprotein (a) in hypothyroid subjects. *Netherlands Journal of Medicine*.

[35] Torun AN, Kulaksizoglu S, Kulaksizoglu M, Pamuk BO, Isbilen E, Tutuncu NB (2009). Serum total antioxidant status and lipid peroxidation marker malondialdehyde levels in overt and subclinical hypothyroidism. *Clinical Endocrinology*.

[36] Becerra A, Bellido D, Luengo A, Piédrola G, De Luis DA (1999). Lippprotein(a) and other lipoproteins in hypothyroid patients before and after thyroid replacement therapy. *Clinical Nutrition*.

[37] Klausen IC, Nielsen FE, Hegedus L, Gerdes LU, Charles P, Faergeman O (1992). Treatment of hypothyroidism reduces low-density lipoproteins but not lipoprotein(a). *Metabolism*.

[38] Ito M, Arishima T, Kudo T (2007). Effect of levo-thyroxine replacement on non-high-density lipoprotein cholesterol in hypothyroid patients. *Journal of Clinical Endocrinology and Metabolism*.

[39] Paoli M, Bellabarba G, Velazquez E (1998). Sex steroids, lipids, and lipoprotein cholesterols in women with subclinical and overt hypothyroidism before and after L-thyroxine therapy. *Clinica Chimica Acta*.

[40] Pazos F, Alvarez JJ, Rubies-Prat J, Varela C, Lasuncion MA (1995). Long term thyroid replacement therapy and levels of lipoprotein(a) and other lipoproteins. *Journal of Clinical Endocrinology and Metabolism*.

[41] Martánez-Triguero ML, Hernández-Mijares A, Nguyen TT (1998). Effect of thyroid hormone replacement on lipoprotein(a), lipids, and apolipoproteins in subjects with hypothyroidism. *Mayo Clinic Proceedings*.

[42] O’Brien T, Katz K, Hodge D, Nguyen TT, Kottke BA, Hay ID (1997). The effect of the treatment of hypothyroidism and hyperthyroidism on plasma lipids and apolipoproteins AI, AII and E. *Clinical Endocrinology*.

[43] Arem R, Escalante DA, Arem N, Morrisett JD, Patsch W (1995). Effect of L-thyroxine therapy on lipoprotein fractions in overt and subclinical hypothyroidism, with special reference to lipoprotein(a). *Metabolism*.

[44] Caraccio N, Ferrannini E, Monzani F (2002). Lipoprotein profile in subclinical hypothyroidism: response to levothyroxine replacement, a randomized placebo-controlled study. *Journal of Clinical Endocrinology and Metabolism*.

[45] Monzani F, Caraccio N, Kozàkowà M (2004). Effect of levothyroxine replacement on lipid profile and intima-media thickness in subclinical hypothyroidism: a double-blind, placebo- controlled study. *Journal of Clinical Endocrinology and Metabolism*.

[46] Efstathiadou Z, Bitsis S, Milionis HJ (2001). Lipid profile in subclinical hypothyroidism: is L-thyroxine substitution beneficial?. *European Journal of Endocrinology*.

[47] Iqbal A, Jorde R, Figenschau Y (2006). Serum lipid levels in relation to serum thyroid-stimulating hormone and the effect of thyroxine treatment on serum lipid levels in subjects with subclinical hypothyroidism: the Tromsø Study. *Journal of Internal Medicine*.

[48] Adrees M, Gibney J, El-Saeity N, Boran G (2009). Effects of 18 months of l-T4 replacement in women with subclinical hypothyroidism. *Clinical Endocrinology*.

[49] Miura S, Iitaka M, Yoshimura H (1994). Disturbed lipid metabolism in patients with subclinical hypothyroidism: effect of L-thyroxine therapy. *Internal Medicine*.

[50] Kung AWC, Pang RWC, Janus ED (1995). Elevated serum lipoprotein(a) in subclinical hypothyroidism. *Clinical Endocrinology*.

[51] Ito M, Takamatsu J, Sasaki I (2004). Disturbed metabolism of remnant lipoproteins in patients with subclinical hypothyroidism. *American Journal of Medicine*.

[52] Ganotakis ES, Mandalaki K, Tampakaki M (2003). Subclinical hypothyroidism and lipid abnormalities in older women attending a vascular disease prevention clinic: effect of thyroid replacement therapy. *Angiology*.

[53] Kim SK, Kim SH, Park KS, Park SW, Cho YW (2009). Regression of the increased common carotid artery-intima media thickness in subclinical hypothyroidism after thyroid hormone replacement. *Endocrine Journal*.

[54] Hueston WJ, Pearson WS (2004). Subclinical hypothyroidism and the risk of hypercholesterolemia. *Annals of Family Medicine*.

[55] Shakoor SKA, Aldibbiat A, Ingoe LE (2010). Endothelial progenitor cells in subclinical hypothyroidism: the effect of thyroid hormone replacement therapy. *Journal of Clinical Endocrinology and Metabolism*.

[56] Bell RJ, Rivera-Woll L, Davison SL, Topliss DJ, Donath S, Davis SR (2007). Well-being, health-related quality of life and cardiovascular disease risk profile in women with subclinical thyroid disease—a community-based study. *Clinical Endocrinology*.

[57] Kvetny J, Heldgaard PE, Bladbjerg EM, Gram J (2004). Subclinical hypothyroidism is associated with a low-grade inflammation, increased triglyceride levels and predicts cardiovascular disease in males below 50 years. *Clinical Endocrinology*.

[58] Yildirimkaya M, Özata M, Yilmaz K, Kilinç C, Gündoğan MA, Kutluay T (1996). Lipoprotein(a) Concentration in Subclinical Hypothyroidism before and after Levo-Thyroxine Therapy. *Endocrine Journal*.

[59] Caron P, Calazel C, Parra HJ, Hoff M, Louvet JP (1990). Decreased HDL cholesterol in subclinical hypothyroidism: the effect of L-thyroxine therapy. *Clinical Endocrinology*.

[60] Lai Y, Wang J, Jiang F (2011). The relationship between serum thyrotropin and components of metabolic syndrome. *Endocrine Journal*.

[61] Milionis HJ, Efstathiadou Z, Tselepis AD (2003). Lipoprotein (a) levels and apolipoprotein (a) isoform size in patients with subclinical hypothyroidism: effect of treatment with levothyroxine. *Thyroid*.

[62] Rondeau G, Rutamucero N, Messier V (2010). Reference range thyroid-stimulating hormone is associated with physical activity energy expenditure in overweight and obese postmenopausal women: a Montreal-Ottawa New Emerging Team Study. *Metabolism*.

[63] Sigal GA, Medeiros-Neto G, Vinagre JC, Diament J, Maranhão RC (2011). Lipid metabolism in subclinical hypothyroidism: plasma kinetics of triglyceride-rich lipoproteins and lipid transfers to high-density lipoprotein before and after levothyroxine treatment. *Thyroid*.

[64] Franklyn JA, Daykin J, Betteridge J (1993). Thyroxine replacement therapy and circulating lipid concentrations. *Clinical Endocrinology*.

[65] Weintraub M, Grosskopf I, Trostanesky Y, Charach G, Rubinstein A, Stern N (1999). Thyroxine replacement therapy enhances clearance of chylomicron remnants in patients with hypothyroidism. *Journal of Clinical Endocrinology and Metabolism*.

[66] Meier C, Staub JJ, Roth CB (2001). TSH-controlled L-thyroxine therapy reduces cholesterol levels and clinical symptoms in subclinical hypothyroidism: a double blind, placebo-controlled trial (basel thyroid study). *Journal of Clinical Endocrinology and Metabolism*.

[67] Arem R, Patsch W (1990). Lipoprotein and apolipoprotein levels in subclinical hypothyroidism. Effect of levothyroxine therapy. *Archives of Internal Medicine*.

[68] Nishitani H, Okamura K, Noguchi S, Inoue K, Morotomi Y, Fujishima M (1990). Serum lipid levels in thyroid dysfunction with special reference to transient elevation during treatment in hyperthyroid graves’ disease. *Hormone and Metabolic Research*.

[69] Guang-Da X, Hong-Yan C, Xian-Mei Z (2004). Changes in endothelium-dependent arterial dilation before and after subtotal thyroidectomy in subjects with hyperthyroidism. *Clinical Endocrinology*.

[70] Muls E, Blaton V, Rosseneu M (1982). Serum lipids and apolipoproteins A-I, A-II, and B in hyperthyroidism before and after treatment. *Journal of Clinical Endocrinology and Metabolism*.

[71] Altinova AE, Törüner FB, Aktürk M (2006). Adiponectin levels and cardiovascular risk factors in hypothyroidism and hyperthyroidism. *Clinical Endocrinology*.

[72] Kung AWC, Pang RWC, Lauder I, Lam KSL, Janus ED (1995). Changes in serum lipoprotein(a) and lipids during treatment of hyperthyroidism. *Clinical Chemistry*.

[73] Klausen IC, Hegedus L, Hansen PS, Nielsen FE, Gerdes LU, Faergeman O (1995). Apolipoprotein(a) phonotypes and lipoprotein(a) concentrations in patients with hyperthyroidism. *Journal of Molecular Medicine*.

[74] Ozata M, Yildirimkaya M, Yilmaz K (1998). The effects of thyroid status on serum apolipoprotein A-I-containing lipoprotein particles. *Hormone and Metabolic Research*.

[75] Hayashi H, Mizushima N, Yoshinaga H (1996). The relationship between lipoprotein(a) and low density lipoprotein receptors during the treatment of hyperthyroidism. *Hormone and Metabolic Research*.

[76] Hoppichler F, Sandholzer C, Moncayo R, Utermann G, Georg Kraft HG (1995). Thyroid hormone (fT4) reduces lipoprotein(a) plasma levels. *Atherosclerosis*.

[77] Berghout A, van de Wetering J, Klootwijk P (2003). Cardiac and metabolic effects in patients who present with a multinodular goitre. *Netherlands Journal of Medicine*.

[78] Parle JV, Franklyn JA, Cross KW, Jones SR, Sheppard MC (1992). Circulating lipids and minor abnormalities of thyroid function. *Clinical Endocrinology*.

[79] Biondi B, Palmieri EA, Fazio S (2000). Endogenous subclinical hyperthyroidism affects quality of life and cardiac morphology and function in young and middle-aged patients. *Journal of Clinical Endocrinology and Metabolism*.

[80] Kotsis V, Alevizaki M, Stabouli S (2007). Hypertension and hypothyroidism: results from an ambulatory blood pressure monitoring study. *Journal of Hypertension*.

[81] Saito I, Ito K, Saruta T (1983). Hypothyroidism as a cause of hypertension. *Hypertension*.

[82] Mayer O, Šimon J, Filipovský J, Pláškova M, Pikner R (2006). Hypothyroidism in coronary heart disease and its relation to selected risk factors. *Vascular Health and Risk Management*.

[83] Christ-Crain M, Meier C, Guglielmetti M (2003). Elevated C-reactive protein and homocysteine values: cardiovascular risk factors in hypothyroidism? A cross-sectional and a double-blind, placebo-controlled trial. *Atherosclerosis*.

[84] Nedrebø BG, Ericsson UB, Nygård O (1998). Plasma total homocysteine levels in hyperthyroid and hypothyroid patients. *Metabolism*.

[85] Morris MS, Bostom AG, Jacques PF, Selhub J, Rosenberg IH (2001). Hyperhomocysteinemia and hypercholesterolemia associated with hypothyroidism in the third US National Health and Nutrition Examination Survey. *Atherosclerosis*.

[86] Squizzato A, Romualdi E, Büller HR, Gerdes VEA (2007). Clinical review: thyroid dysfunction and effects on coagulation and fibrinolysis: a systematic review. *Journal of Clinical Endocrinology and Metabolism*.

[87] Erem C (2009). Coagulation and fibrinolysis in thyroid dysfunction. *Endocrine*.

[88] Chadarevian R, Bruckert E, Leenhardt L, Giral P, Ankri A, Turpin G (2001). Components of the fibrinolytic system are differently altered in moderate and severe hypothyroidism. *Journal of Clinical Endocrinology and Metabolism*.

[89] Erdogan M, Canataroglu A, Ganidagli S, Kulaksızoglu M Metabolic syndrome prevelance in subclinic and overt hypothyroid patients and the relation among metabolic syndrome parameters.

[90] Dimitriadis G, Mitrou P, Lambadiari V (2006). Insulin action in adipose tissue and muscle in hypothyroidism. *Journal of Clinical Endocrinology and Metabolism*.

[91] Giménez-Palop O, Giménez-Pérez G, Mauricio D (2005). Circulating ghrelin in thyroid dysfunction is related to insulin resistance and not to hunger, food intake or anthropometric changes. *European Journal of Endocrinology*.

[92] Caixàs A, Tirado R, Vendrell J (2009). Plasma visfatin concentrations increase in both hyper and hypothyroid subjects after normalization of thyroid function and are not related to insulin resistance, anthropometric or inflammatory parameters. *Clinical Endocrinology*.

[93] Dimitriadis G, Maratou E, Boutati E (2008). IGF-I increases the recruitment of GLUT4 and GLUT3 glucose transporters on cell surface in hyperthyroidism. *European Journal of Endocrinology*.

[94] Dimitriadis G, Parry-Billings M, Bevan S (1997). The effects of insulin on transport and metabolism of glucose in skeletal muscle from hyperthyroid and hypothyroid rats. *European Journal of Clinical Investigation*.

[95] Çakal E, Turgut AT, Demirbas B (2009). Effects of L-thyroxine replacement therapy on carotid intima-media thickness in patients with primary hypothyroidism. *Experimental and Clinical Endocrinology and Diabetes*.

[96] Nagasaki T, Inaba M, Kumeda Y (2005). Decrease of arterial stiffness at common carotid artery in hypothyroid patients by normalization of thyroid function. *Biomedicine and Pharmacotherapy*.

[97] Vanhaelst L, Neve P, Bastenie PA (1967). Coronary-artery disease in myxoedema. *The Lancet*.

[98] Steinberg AD (1968). Myxedema and coronary artery disease–a comparative autopsy study. *Annals of Internal Medicine*.

[99] Qureshi AI, Suri MFK, Nasar A, Kirmani JF, Divani AA, Giles WH (2006). Free Thyroxine Index and risk of stroke: results from the National Health and Nutrition Examination Survey follow-up study. *Medical Science Monitor*.

[100] Liu D, Jiang F, Shan Z (2010). A cross-sectional survey of relationship between serum TSH level and blood pressure. *Journal of Human Hypertension*.

[101] Nagasaki T, Inaba M, Kumeda Y (2006). Increased pulse wave velocity in subclinical hypothyroidism. *Journal of Clinical Endocrinology and Metabolism*.

[102] Duan Y, Peng W, Wang X (2009). Community-based study of the association of subclinical thyroid dysfunction with blood pressure. *Endocrine*.

[103] Walsh JP, Bremner AP, Bulsara MK (2006). Subclinical thyroid dysfunction and blood pressure: a community-based study. *Clinical Endocrinology*.

[104] Toruner F, Altinova AE, Karakoc A (2008). Risk factors for cardiovascular disease in patients with subclinical hypothyroidism. *Advances in Therapy*.

[105] Şengul E, Çetinarslan B, Tarkun I, Cantürk Z, Türemen E (2004). Homocysteine concentrations in subclinical hypothyroidism. *Endocrine Research*.

[106] Lindeman RD, Romero LJ, Schade DS, Wayne S, Baumgartner RN, Garry PJ (2003). Impact of subclinical hypothyroidism on serum total homocysteine concentrations, the prevalence of coronary heart disease (CHD), and CHD risk factors in the New Mexico Elder Health Survey. *Thyroid*.

[107] Hueston WJ, King DE, Geesey ME (2005). Serum biomarkers for cardiovascular inflammation in subclinical hypothyroidism. *Clinical Endocrinology*.

[108] Luboshitzky R, Aviv A, Herer P, Lavie L (2002). Risk factors for cardiovascular disease in women with subclinical hypothyroidism. *Thyroid*.

[109] Cappola AR, Fried LP, Arnold AM (2006). Thyroid status, cardiovascular risk, and mortality in older adults. *Journal of the American Medical Association*.

[110] Jung CH, Sung KC, Shin HS (2003). Thyroid dysfunction and their relation to cardiovascular risk factors such as lipid profile, hsCRP, and waist hip ratio in Korea. *The Korean Journal of Internal Medicine*.

[111] Tuzcu A, Bahceci M, Gokalp D, Tuzun Y, Gunes K (2005). Subclinical hypothyroidism may be associated with elevated high-sensitive C-reactive protein (low grade inflammation) and fasting hyperinsulinemia. *Endocrine Journal*.

[112] Razvi S, Weaver JU, Vanderpump MP, Pearce SHS (2010). The incidence of ischemic heart disease and mortality in people with subclinical hypothyroidism: reanalysis of the Whickham survey cohort. *Journal of Clinical Endocrinology and Metabolism*.

[113] Singh S, Duggal J, Molnar J, Maldonado F, Barsano CP, Arora R (2008). Impact of subclinical thyroid disorders on coronary heart disease, cardiovascular and all-cause mortality: a meta-analysis. *International Journal of Cardiology*.

[114] Rodondi N, Aujesky D, Vittinghoff E, Cornuz J, Bauer DC (2006). Subclinical hypothyroidism and the risk of coronary heart disease: a meta-analysis. *American Journal of Medicine*.

[115] Ochs N, Auer R, Bauer DC (2008). Meta-analysis: subclinical thyroid dysfunction and the risk for coronary heart disease and mortality. *Annals of Internal Medicine*.

[116] Razvi S, Shakoor A, Vanderpump M, Weaver JU, Pearce SHS (2008). The influence of age on the relationship between subclinical hypothyroidism and ischemic heart disease: a metaanalysis. *Journal of Clinical Endocrinology and Metabolism*.

[117] Goksel BK, Karatas M, Nebioglu A (2007). Subclinical hypothyroidism, hyperhomocysteinemia and dyslipidemia: investigating links with ischemic stroke in Turkish patients. *Neurological Research*.

[118] Jeong SK, Seo JY, Nam HS, Park HK (2010). Thyroid function and internal carotid artery stenosis in ischemic stroke. *Endocrine Journal*.

[119] Alevizaki M, Synetou M, Xynos K, Alevizaki CC, Vemmos KN (2006). Hypothyroidism as a protective factor in acute stroke patients. *Clinical Endocrinology*.

[120] Baek JH, Chung PW, Kim YB (2010). Favorable influence of subclinical hypothyroidism on the functional outcomes in stroke patients. *Endocrine Journal*.

[121] Rodondi N, Newman AB, Vittinghoff E (2005). Subclinical hypothyroidism and the risk of heart failure, other cardiovascular events, and death. *Archives of Internal Medicine*.

[122] Prisant LM, Gujral JS, Mulloy AL (2006). Hyperthyroidism: a secondary cause of isolated systolic hypertension. *Journal of Clinical Hypertension*.

[123] Colleran KM, Ratliff DM, Burge MR (2003). Potential association of thyrotoxicosis with vitamin B and folate deficiencies, resulting in risk for hyperhomocysteinemia and subsequent thromboembolic events. *Endocrine Practice*.

[124] Mitrou P, Boutati E, Lambadiari V (2010). Insulin resistance in hyperthyroidism: the role of IL6 and TNF*α*. *European Journal of Endocrinology*.

[125] Dimitriadis G, Mitrou P, Lambadiari V (2006). Glucose and lipid fluxes in the adipose tissue after meal ingestion in hyperthyroidism. *Journal of Clinical Endocrinology and Metabolism*.

[126] Al Jaber J, Haque S, Noor H, Ibrahim B, Al Suwaidi J (2010). Thyrotoxicosis and coronary artery spasm: case report and review of the literature. *Angiology*.

[127] Chudleigh RA, Davies JS (2007). Graves’ thyrotoxicosis and coronary artery spasm. *Postgraduate Medical Journal*.

[128] Choi YH, Chung JH, Bae SW (2005). Severe coronary artery spasm can be associated with hyperthyroidism. *Coronary Artery Disease*.

[129] Sheu JJ, Kang JH, Lin HC, Lin HC (2010). Hyperthyroidism and risk of ischemic stroke in young adults: a 5-year follow-up study. *Stroke*.

[130] Staffurth JS, Gibberd MC, Fui SN (1977). Arterial embolism in thyrotoxicosis with atrial fibrillation. *British Medical Journal*.

[131] Bar-Sela S, Ehrenfeld M, Eliakim M (1981). Arterial embolism in thyrotoxicosis with atrial fibrillation. *Archives of Internal Medicine*.

[132] Squizzato A, Gerdes VEA, Brandjes DPM, Büller HR, Stam J (2005). Thyroid diseases and cerebrovascular disease. *Stroke*.

[133] Völzke H, Ittermann T, Schmidt CO (2009). Subclinical hyperthyroidism and blood pressure in a population-based prospective cohort study. *European Journal of Endocrinology*.

[134] Völzke H, Robinson DM, Schminke U (2004). Thyroid function and carotid wall thickness. *Journal of Clinical Endocrinology and Metabolism*.

